# Plant‐based oral vaccines against zoonotic and non‐zoonotic diseases

**DOI:** 10.1111/pbi.12604

**Published:** 2016-08-23

**Authors:** Naila Shahid, Henry Daniell

**Affiliations:** ^1^ Department of Biochemistry School of Dental Medicine University of Pennsylvania Philadelphia PA USA

**Keywords:** Livestock, molecular farming, oral delivery, animal diseases, veterinary vaccines

## Abstract

The shared diseases between animals and humans are known as zoonotic diseases and spread infectious diseases among humans. Zoonotic diseases are not only a major burden to livestock industry but also threaten humans accounting for >60% cases of human illness. About 75% of emerging infectious diseases in humans have been reported to originate from zoonotic pathogens. Because antibiotics are frequently used to protect livestock from bacterial diseases, the development of antibiotic‐resistant strains of epidemic and zoonotic pathogens is now a major concern. Live attenuated and killed vaccines are the only option to control these infectious diseases and this approach has been used since 1890. However, major problems with this approach include high cost and injectable vaccines is impractical for >20 billion poultry animals or fish in aquaculture. Plants offer an attractive and affordable platform for vaccines against animal diseases because of their low cost, and they are free of attenuated pathogens and cold chain requirement. Therefore, several plant‐based vaccines against human and animals diseases have been developed recently that undergo clinical and regulatory approval. Plant‐based vaccines serve as ideal booster vaccines that could eliminate multiple boosters of attenuated bacteria or viruses, but requirement of injectable priming with adjuvant is a current limitation. So, new approaches like oral vaccines are needed to overcome this challenge. In this review, we discuss the progress made in plant‐based vaccines against zoonotic or other animal diseases and future challenges in advancing this field.

## Introduction

Zoonosis can be defined as transmission of disease between human and animals that happens due to interaction between these two populations. Zoonosis not only interrupts human health but it also affects wild life and livestock industry. Recently, more than 65% of emerging infectious diseases in humans have been reported to originate from zoonotic pathogens (Narrod *et al*., 2012). Zoonotic diseases can be divided into two categories depending on their mode of transmission. Vector‐borne zoonotic infection is transmitted to humans via arthropods carrier and nonvector‐borne diseases are transferred by contaminated food or direct contact (Buza *et al*., 2015). Zoonotic infections in humans started 14 000 years ago when dogs were domesticated for hunting. Other animals like goat, sheep, cattle and pigs were domesticated later for meat or dairy products. This domestication increased not only the interaction between these two populations but also the risk of their cross‐contaminating diseases (Thrusfield, 2013).

The plague outbreak that killed a large proportion of the European population was spread through rats and is an example of the profound impact of zoonotic diseases. After smallpox, plague is historically the second most deadly disease in human populations and has caused millions of deaths; the Black Death pandemic in Europe during the 14th–17th centuries caused 200 million deaths and killed one‐third of European population (Alvarez and Cardineau, 2010; Perry and Fetherston, 1997). According to an estimate, 31 food‐borne pathogens of animal origin in United States cause 9.4 million cases of illness each year. Salmonella spp., norovirus, Campylobacter spp, Toxoplasma gonidii, avian influenza virus, swine flu virus, Listeria monocytogenes and norovirus are major pathogens, producing zoonotic infections in humans. Salmonella is a major human health concern in the United States, causing one million cases of illness and 400 deaths annually. Salmonella is the most common pathogen in turkey and poultry and causes multiple human salmonellosis in the United States through contaminated food (Routh *et al*., 2015). Norovirus is another important zoonotic pathogen in the United States. It is responsible for 50% of food‐borne gastroenteritis outbreaks in the United States, leading to 19–21 million cases of illness and 570–800 deaths annually. It is a major cause of viral gastrointestinal outbreaks in cruise ships in the USA and spreads by direct contact. Contaminated food is an another important source for the spread of norovirus infection in the United States (Stock *et al*., 2015). Similarly in Australia 30% illness is caused by food‐borne pathogens and in England and Wales 8% illness is due to contaminated food of animal origin by Norovirus. Moreover, people working in production and farm areas are at high risk of acquiring infection from various animals. Farm workers act as channel to spread zoonotic pathogens to the public (Gray and Kayali, 2009). Although zoonotic diseases kill a much larger population in developing countries, accurate documentation or statistics is not available.

Growing populations and increased demand for food have led to increased production of food of animal origin. In most developing countries, almost 66% of the population is protein deficient; the total protein requirement per person is 103 g per day, but protein‐deficient individuals consume only 69% of this amount, most of it in the form of red and white meat. Livestock production has played a major role in meeting this demand. Among livestock, ruminants and poultry are the cheapest and most efficient source of high‐quality protein; they can easily compensate for protein deficiencies among the population (Ashraf and Shah, 2014). Livestock production is playing very important role in accomplishing food security, influenced strongly by cultural preferences (Godber and Wall, 2014). According to the United States Department of Agriculture (USDA), current global beef and veal production is almost 59.2 million tons and is expected to increase by 1% in 2016. Pork production is now at 112.0 million tons and is expected to remain stable in 2016, and 89.3 million tons of broiler meat is produced and is expected to rise 2% in 2016. The United States and India are largest producers of beef, whereas Australia is the largest beef supplier. Similarly, the United States, Brazil and China are largest producers of broiler meat (http://www.usda.gov/wps/portal/usda/usdahome). Animals support life on earth by providing high‐quality protein through dairy products and meat, but this industry is severely hampered by parasitic and infectious diseases of both endemic and epidemic in nature, negatively impacting global economy (Rich and Perry, 2011). For example, the epidemic outbreak of foot‐and‐mouth disease in the United Kingdom in 2001 resulted in direct economic loss of $11.9–$18.4 billion including loss of agriculture and food industry (Carpenter *et al*., 2011). Animal health is an important element in providing safe products to humans. Animal disease management is a major concern for livestock, poultry and fish industries to provide pathogen‐free products to the consumer (Kolotilin *et al*., 2014).

The complex global system and human interaction with pet animal, livestock and poultry has made it essential to consider human and animal health as a common and not two isolated problems. However, development of medicines to control animal diseases is a big challenge in field of veterinary medicine. The major challenges are the cost and volume. Most human vaccines are given as injections and it is impossible to inject large numbers of animals. Lives of farmed animals are very different from their natural lives, as most animals in the United States are reared in factory farms. This favours increased production and maximizes profit margins but also increases the risk of the spread of infectious diseases. Live attenuated and killed vaccines are the only available option to control these infectious diseases. However, the main problem with them is their high cost versus profit, and the use of injectable vaccines is impractical, if not impossible, to control disease in large farms.

Plants offer an attractive and affordable platform for vaccines against animal diseases, especially in industries with low profit margins. So, edible orally delivered, low‐cost vaccines are an urgent need in the production of disease‐free animals. Topp *et al*. (2016) recently provided a general overview of efficacy, competitiveness and regulatory approval of plant‐made veterinary immunotherapeutics. This manuscript is a highly comprehensive in‐depth review of plant‐made veterinary vaccines to control zoonotic diseases, which has not been addressed in any previous review. In addition, mechanisms of different vaccine delivery methods and vaccines to control nonzoonotic diseases are also reviewed.

### Current strategies to control zoonotic and animal diseases

The first discovery of small pox vaccine by Edward Jenner opened a new world for the prevention of infectious diseases. Since that time, many approaches have been adopted to control humans and animal diseases using essentially the same concept (Joensuu *et al*., 2008). Behring and Kitasato first introduced the concept of serum therapy when they induced immunity in animals by serum treated with nonlethal toxin dose (Behring *et al*., 1890). Later on, advancement in proteomics resulted in the use of mouse hybridoma cells from immunized mice to produce specific monoclonal therapeutic antibodies (Köhler and Milstein, 1975) that conferred protection against human diseases. But use of antibody therapy is severely limited by their high production cost. The estimated high production cost for protein drug ($140 billion in 2013) has made them unaffordable in most developing countries, as most people earn less than $2 per day (Kwon and Daniell, 2015). Infectious diseases of animals are a large concern throughout the world, especially in developing countries. It is estimated that 58% human pathogens are zoonotic, that is transmitted from animals, major cause for emerging infectious diseases. Vaccination is the only possible tool to control these infectious diseases especially in animals with short life span (Loza‐Rubio and Rojas‐Anaya, 2010).

Since 1940, a number of vaccines have been developed using inactivated, attenuated and live viruses. The most common and licensed vaccines against infectious diseases of animals are live attenuated or killed live attenuated pathogens or recombinant proteins. Porcilis‐PCV2 and Suvaxyn PCV2 for pigs, Periovac for dogs, AquaVac ERM, AquaVac Furuvac, AquaVac Vibrio for fish are commercialized and licensed vaccines against veterinary diseases (Meeusen *et al*., 2007). In inactivated virus‐based vaccines, the disease‐causing virus has been killed by heat, radiation or other methods. The drawback of this type of vaccine is that it causes a weaker immune response than live vaccines and several booster doses are needed to acquire an adequate level of immunity. Live vaccines contain live virus that is weak but still infectious. Live virus‐based vaccines are usually prepared in lyophilized form and can be stored for up to 1 year at 4 °C. PreveNile against horses, Vaxxitek HVT+IBD against poultry, Bovilis IBR Marker against cattle, RECOMBITEK Canine Parvo against dogs, RECOMBITEK Corona MLV against dogs, Enterisol Ileitis against pigs are commercially available live virus vaccines (Meeusen *et al*., 2007). Live virus vaccines have some disadvantages, as there is always a risk of their regaining virulence. Although these are not well documented in veterinary vaccines because of their short life, they are well documented in human vaccines. Sabin strains used in oral polio vaccine (OPV) revert to virulence by recombination with other enteroviruses or by point mutations (Burns *et al*., 2014; Runckel *et al*., 2013). Several decades ago, genetic instability and vaccine‐associated paralytic poliomyelitis (VAPP) was reported among recipients of OPV in the United States and their close contacts (Alexander *et al*., 2004; Schonberger *et al*., 1976). Outbreaks in the Dominican Republic and Haiti a decade ago (Kew *et al*., 2002) led to the discovery of circulating vaccine‐derived polioviruses (VDPV) (Diop *et al*., 2015; Lakhani and Bumb, 2014). Therefore, the World Health Organization has recommended complete withdrawal of OPV Type 2 by April 2016 globally (Chan *et al*., 2016). Such an outbreak of attenuated live vaccines against zoonotic diseases is a major concern. In addition, the mode of delivery of live vaccines via sprays or aerosols pose additional challenges. The aerosol mode of administration can unintentionally inoculate younger or more susceptible animals, which can ultimately cause death (Alexander, 2012).

Advances in biotechnology have resulted in the development of recombinant therapeutics. *E. coli* was used as first expression system for the production of recombinant therapeutics. After a number of efforts in the field of recombinant therapeutics and approval of *Escherichia coli‐*expressed recombinant human insulin established the importance of recombinant therapeutics. Recombinant therapeutics production involves the expression and purification of immunogenic antigens instead of the whole virus or pathogens. These recombinant vaccines are useful to control infectious diseases of animals due to reduced risk associated with live and killed viral vaccines. Recombinant therapeutics are expensive to produce but they are found to be effective in humans. There are different systems to produce recombinant therapeutics, including bacteria, insect cells, yeast, mammalian cell culture and transgenic animals. Bacteria and yeast‐based production systems are more efficient because of their rapid replication rate. However, recombinant proteins need to be purified to remove host‐derived proteins, and this is a major contributing factor to the cost of recombinant therapeutics (Joensuu *et al*., 2008). The global market of animal vaccine is almost $5507.3 million and it is likely to increase $7197.9 million in 2020. The animal vaccine market is based on poultry diseases, livestock diseases, porcine diseases, aquaculture and equine diseases. The expansion of vaccine market is boosted by many factors, but the main factor is increasing incidence of zoonotic infections in humans, which spreads by direct contact with animals or food‐borne pathogens (Kolotilin *et al*., 2014). Expression of protective antigens in plants is a new vaccination technology that is more economical than any other available system of vaccines production (Joensuu *et al*., 2008).

### Plant‐made vaccines against zoonotic and animal diseases

Edible vaccine production for veterinary use has received widespread attention because of health initiatives aimed at decreasing antibiotic use in livestock and other animals to avoid the development of antibiotic‐resistant strains, especially of epidemic and zoonotic pathogens. These issues have promoted the development of plant‐based vaccines, which can easily fulfil these requirements (Sack *et al*., 2015). Various vaccines against infectious animal diseases are available in the market and show good results, but they have several disadvantages (Meeusen *et al*., 2007). The major drawbacks of these vaccines are their complex and expensive production and purification, their requirement for low‐temperature storage, safety issues and the need for a skilled person for administration. Subunit vaccines are expensive and difficult to produce and always requires low‐temperature storage. Apex‐IHN against Salmon and West Nile‐Innovator DNA against horses are commercially available DNA vaccines, but major drawbacks of DNA vaccines are monotonic responses and require extensive safety protocols (Meeusen *et al*., 2007). Whole‐cell vaccines also have problems associated with administration and low‐temperature storage. Another main reason to avoid conventional vaccine for most of viral infection is that their use can affect disease‐free status of country by enhancing disease scrutiny. For example, inactivated vaccine for FMD is quite effective but it is still banned in disease‐free countries because of its consequence on international trade (Ruiz *et al*., 2015).

The field of plant genetic engineering started in 1970, when extensive research was carried out to discover ways to utilize plant genetics for reasons other than nutrition. After early studies on expression of a few biopharmaceuticals, antigens against different human and animal diseases were expressed in plants (Liew and Hair‐Bejo, 2015). The United States Department of Agriculture (USDA) approved the world's first plant‐based vaccine in 2006. Dow AgroSciences received approval for the first plant‐based vaccine against Newcastle disease virus (NDV) from the USDA. Dow Agro Sciences used tobacco suspension cell lines to develop a plant‐based vaccine (injectable) against NDV. This vaccine was approved by the USDA in 2006 after showing 90% protection against a challenge with NDV virus. This system can be used to produce a large quantity of antigen in a very short period of a few weeks, but the company did not commercialize the product. Although this is a plant‐based vaccine, injectable mode of administration did not offer significant cost advantages. However, the success of the first commercial plant‐made vaccine against NDV by Dow Agro Sciences opened the door for the commercialization of plant‐made vaccines (Yusibov *et al*., 2011).

**Figure 1:**
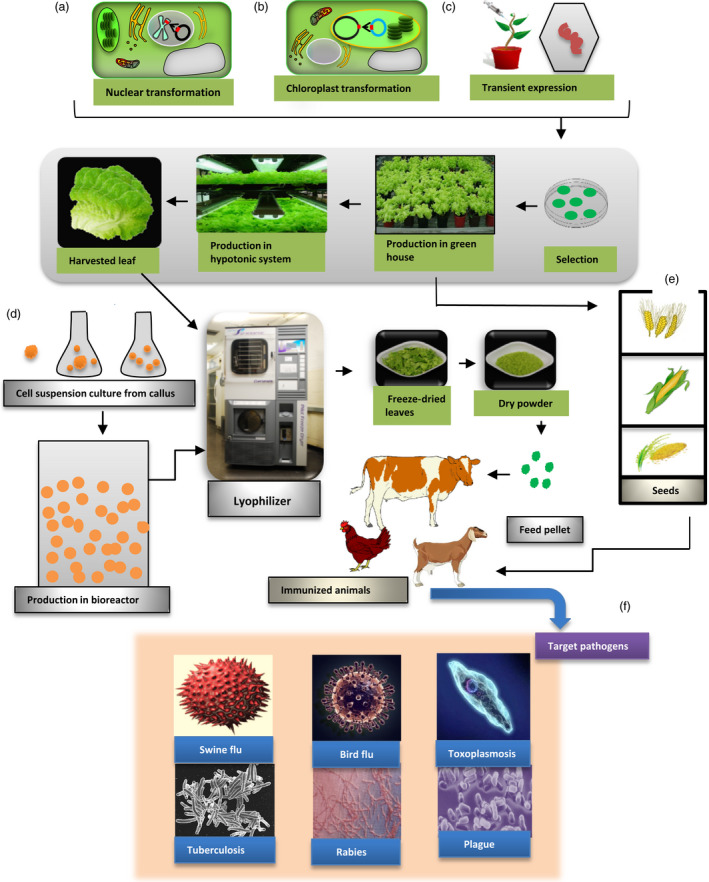
Mechanism for production of plant‐based oral vaccines against animal diseases. (a) Nuclear expression of foreign antigens via *Agrobacterium tumefaciens‐mediated transformation* followed by selection and propagation in the glasshouse. (b) In chloroplast transformation, a transgene is introduced into the plant chloroplast genome using a particle gun, resulting in site‐specific integration by homologous recombination. (c) Transient expression system based on engineered virus and *Agrobacterium tumefaciens* to express foreign antigens in plant cells. (d) Cell suspension culture derived from transformed calli expressing vaccine antigens. (e) Harvested leaves are lyophilized to produce dry powder that can be stored for many years without losing efficacy of expressed vaccine antigens. Orally immunization of animals with feed pellets or seeds from transgenic plants for immunization against target pathogens.

Edible vaccines are actually recombinant vaccines in which selected antigens against a particular pathogen are introduced into a plant. Oral delivery of this plant induces a protective immune response against that particular pathogen in the form of an edible vaccine (Aswathi *et al*., 2014). Almost 200 proteins have been produced in plants, and their promising results make them new competitors in the field of recombinant proteins. Vaccine production through plants has several advantages over other eukaryotic production systems. They are cost‐effective and safe and can be produced in large quantities. With plant‐based production systems, a glasshouse or a plot of land can easily replace the expensive use of fermenters (Daniell *et al*., 2016b, 2009). The estimated cost for plant‐based production is much lower as compared to mammalian and *E. coli* system with basic need of light, water and simple large‐scale production method. Vaccine antigen can be delivered easily just in the form of plant material instead of needle‐based delivery (Liew and Hair‐Bejo, 2015). Plant‐based medicines are usually free from the toxins and pathogens that are commonly produced in bacteria and yeast (Kwon *et al*., 2013). Plant cells expressing vaccine antigens or biopharmaceuticals can be lyophilized and stored at ambient temperature for many years maintaining efficacy of expressed protein drugs (Kwon *et al*., 2013; Lakshmi *et al*., 2013; Su *et al*., 2015; Kwon *et al*., 2013; Lakshmi *et al*., 2013; Su *et al*., 2015). Except for their lower cost, plant‐produced proteins and therapeutics are very similar to recombinant proteins that have been produced in eukaryotes (Daniell *et al*., 2009). Plant‐based vaccines or therapeutic proteins can be post‐translationally modified. Moreover, glycans are different in plants and animals and this has a strong effect on immunogenicity (Chan and Daniell, 2015; Kwon and Daniell, 2015; Tremblay *et al*., 2010). They also negate the issue of restored virulence that is associated with live vaccines (Clarke *et al*., 2013). Plant‐based vaccines have the potential to induce a mucosal immune response and a systemic immune response without the pain and risk associated with needles and injections. Another advantage of plant‐based vaccines is that they can be given directly to animals after oral priming with adjuvants (Chan and Daniell, 2015). The plant cell wall protects the foreign antigen until it is digested by gut microbes, releasing the antigen and promoting the immune response (Kwon *et al*., 2013). Animal studies show that these vaccines are protected from degradation by the digestive enzymes of the stomach through bioencapsulation, and they can produce a protective immune response after encountering a pathogen (Figure 1) (Lakshmi *et al*., 2013). The best plants for edible vaccines are vegetables and fruits like potato, tomato, carrot, maize, banana, lettuce, tobacco, and rice and soya bean. However, it is difficult to control antigen dose in fresh fruits and vegetables. Therefore, plant material should be freeze‐dried to control dose (Chan and Daniell, 2015; Daniell *et al*., 2016a). When animals eat transgenic seeds, fruit or plants in the form of edible vaccines, mucosal‐specific antibodies (Okamura *et al.,* 2014) and serum‐specific antibodies (IgG) are produced (Sack *et al*., 2015). In most animals and birds, the gut‐associated lymphoid tissue (GALT) is responsible for inducing an immune response (Aswathi *et al*., 2014). Antigens from the lumen or intestine are recognized by M cells in the Peyer's patches, and M cells activate B lymphocytes with assistance of Th cells. B lymphocytes produce the mucosal immune response and IgA, which kills pathogens by binding to surface proteins. Another method of antigen uptake is mediated by dendritic intestinal cells, which induce a systemic immune response in the form of IgG antibodies. Cytotoxic T lymphocytes (CTL) are activated by Th cells and induce a strong cellular immune response. Thus, edible vaccines have the potential to induce mucosal, systemic and cellular immune responses (Chan and Daniell, 2015; Guan *et al*., 2013).

### Plant expression systems to produce vaccine antigens

#### Nuclear transformation

Nuclear transformation is the simplest and most extensively used system for the production of genetically modified crops. In this system, a foreign antigen is expressed from the nuclear genome via *Agrobacterium tumefaciens* or biolistic gene gun‐mediated transformation (Figure 1a) *Agrobacterium tumefaciens* is a soil‐borne, Gram‐negative bacterium that can transform a foreign gene into a host by making a crown gall. *Agrobacterium* infects plant tissues by sensing the phenolic secretions of wounded plants. These specific signals activate bacterial virulence (Vir) genes that produce Vir proteins. Ti plasmids induce the formation of T‐DNA molecules. T‐DNA associates with Vir proteins to make a T‐DNA complex. A complex network between bacterial Vir proteins and T‐DNA ultimately transfers the T‐DNA into the nuclear genome of the host plant. The T‐DNA principle of gene transfer has made it an important tool in plant genetic engineering to transform foreign genes into plants (Kim and Yang, 2010; Pitzschke, 2013). Foreign genes integrate into the nuclear genome, allowing the continuous production of recombinant protein (Chan and Daniell, 2015; Guan *et al*., 2013; Tremblay *et al*., 2010). Another advantage of nuclear transformation is the post‐translational modification of recombinant proteins that occurs in this eukaryotic production system. After post‐translational modification, depending on signal peptides, proteins can be stored in various organelles or secreted (Tremblay *et al*., 2010). But there are several disadvantages linked with this system including gene silencing, position effect, low expression level and risk of transgene contamination via pollen or seeds, limiting commercial development of plant‐based recombinant vaccines (Fahad *et al*., 2015). A few transgenic plant‐based vaccines have moved forward to clinical trials, but none of them reached beyond phase I clinical trials, mainly due to low expression level that limited their regular approval from FDA. Expressed norovirus capsid protein VP1 in potato tubers against norovirus has been advanced to phase I clinical trials where 20% vaccinated volunteers produced IgG in titre range 1 : 757. It was prepared by Arizona State University in collaboration with GMP facility (Takeyama *et al*., 2015). Similarly edible vaccines against entertoxigenic *E. coli* in potato and maize have advanced to phase I clinical trials where vaccinated volunteers produced LTB‐specific IgG and IgA. Another, rice‐based cholera vaccine that was developed in Japan is now under phase I clinical trials (Takeyama *et al*., 2015).

#### Chloroplast transformation

Chloroplast transformation addresses some of the problems of nuclear transformation for commercialization of plant‐based recombinant vaccines. In this method, a transgene is introduced into the circular plant chloroplast genome through a particle gun, resulting in site‐specific integration by homologous recombination (Figure 1b) (Daniell *et al*., 2002). In this method, when leaves are bombarded with gold particles coated with chloroplast vectors, transgene cassette integrates into the chloroplast genome. The expression of foreign genes is generally high, as there are 10 000 copies of the chloroplast genome in each leaf cell. As chloroplast genome is maternally inherited, this reduces the risk of transgene escape via pollen. In addition, harvesting of vegetative tissues (leaves) before flowering eliminates escape via pollen or seeds. Therefore, transplastomic plants expressing vaccine antigens and biopharmaceuticals have been grown in the field (Arlen *et al*. 2007). USDA‐APHIS has certified that transplastomic lines do not fit the definition of a regulated article 7 CFR (part 340) because there are no pest components (Kwon and Daniell, 2015). Moreover, expression of multiple gene is possible under a single promoter due to polycistronic expression (De Cosa *et al*. 2001; Quesada‐Vargas *et al*. 2005). The major regulatory element used in chloroplast transformation is the *psbA* promoter, 5′ untranslated region (UTR), *psbA* 3′ untranslated region and the most commonly used spacer region is the trn1 and trnA (Daniell *et al*., 2016a,b). Such combination facilitates very high levels of transgene expression, even up to 72% of TSP (Chan and Daniell, 2015). Many antigens from viral and bacterial origin has been expressed in chloroplast against different animals diseases including polio, plague, cholera, malaria, canine parvovirus, tuberculosis, anthrax, FMD, rotavirus, classical swine flu virus (Arlen *et al*., 2008; Gorantala *et al*., 2014; Lakshmi *et al*., 2013; Lentz *et al*., 2010; Ortigosa *et al*., 2010; Shao *et al*., 2008; Zhou *et al*., 2010).

### Viral vectors

Another transformation system uses plant viral vectors like cauliflower mosaic virus (CaMV), tobacco mosaic virus, cowpea mosaic virus, bamboo mosaic virus or alfalfa mosaic virus (Figure 1c). In this method, the plant virus is genetically engineered to be under the control of the coat protein subgenomic mRNA promoter. The coat protein is highly expressed in an infected host, making it the best promoter to express foreign genes. Copies of the infectious nucleic acid deliver the target gene to the plant cell. They produce virus‐like particles that deliver the peptide epitope. This process of infecting most plant tissues takes almost 3 weeks (Gleba *et al*., 2007). The plant virus system has resulted in the production of vaccines against rabies in which modified alfalfa mosaic virus CP glycoprotein G and the nucleoprotein were expressed by the viral vector system and conferred protection in mice after viral challenge. Plant‐based rabies vaccines is in phase I clinical trials; five of nine volunteers produced neutralizing antibodies against rabies virus (Rybicki, 2014; Takeyama *et al*., 2015). Plant viruses are independently transcribed and translated to produce abundant proteins in a short time (Guan *et al*., 2013).

In plant virus and agrobacterium based transformation, *Agrobacterium* promotes the entry of multiple copies of recombinant viral vectors into plant cells. The target gene is delivered through *Agrobacterium* by one of two methods: injecting the stomata of leaves or vacuum infiltration. In vacuum infiltration, upper parts of a plant are flooded with a culture of *Agrobacterium* and a vacuum is applied to remove air from intracellular spaces. Upon release, the vacuum delivers the target gene to plant cells via *Agrobacterium* (Ling *et al*., 2010). Transcripts of the recombinant viral genome enter the nucleus and cytoplasm of plant cells. As a result, plants start producing large quantities of the target protein within a few days (Yusibov and Rabindran, 2008). This transient expression system has produced target proteins against *Bacillus anthracis, Yersinia pestis* and influenza virus (Chichester *et al*., 2007; Shoji *et al*., 2008). Transient expression system also resulted in the production of different veterinary vaccines against bluetongue virus, Crimean‐Congo haemorrhagic fever virus, Ebola virus, Rift Valley fever virus with promising results (Rybicki, 2014).

Transient expression system with both viral vector‐ and agrobacterium‐mediated delivery of virus replicon is the most preferred method to achieve high level expression of foreign genes. Transient expression system has produced a number of medically important antigens against different diseases. The magnICON technology is also a modification in transient expression to enhance gene expression. Ma *et al*. (2012) used modified magnICON tobacco mosaic virus‐based transient expression system to produce plant‐made PyMSP119 against malaria. The highest expression level reached up to 23% of TSP (Fahad *et al*., 2015; Ma *et al*., 2012). Larsen and Curtis (2012) used replicating PVX vectors and a nonreplicating CPMV‐HT vector and achieved the highest expression of heterologous protein in tobacco hairy roots almost 27.6% of TSP (Larsen and Curtis, 2012).

### Cell suspension cultures

Cell suspensions are individual cells or cell aggregates that are derivatives of callus tissues; separated callus cells propagate to produce a stable cell suspension (Figure 1d). Transgenic explants or a single callus cell can produce recombinant antigens via transformation with *Agrobacterium* and can then be easily scaled up in a fermenter. In 2006, the USDA approved the world's first edible vaccine against poultry diseases, which was prepared in tobacco cell suspension (Yusibov and Rabindran, 2008). In 2012, FDA approved the first biopharmaceutical for protein made in carrot cells—glucocerebrosidase to treat Gaucher's disease. This protein was developed by an Israeli company Protalix Biotherapeutics, and FDA‐approved product is now marketed by Pfizer (Wolfson, 2013).

### Strategies to enhance expression of vaccine antigens

In early work on edible vaccines, the expression level of expressed antigen was not high, for example rotavirus VP6 protein in potatoes produced low expression 0.02% of TSP (Matsumura *et al*., 2002) and infectious bronchitis S1 protein in potatoes resulted in low expression 0.07%–0.22% of TSP (Zhou *et al*., 2003). Therefore, low expression level was a major limiting factor in the field of plant vaccines. Different strategies have been adopted to increase expression of transformed genes. In particular, extensive effort has been made to improve expression of transgenes to make edible vaccines more immunogenic. One of the most common approaches is the use of suitable promoters. The CaMV 35S promoter is constitutive and is used in dicotyledonous plants. It is the promoter of choice for expressing foreign antigens in all parts of most dicotyledonous plants. The ubiquitin promoter is commonly used in monocotyledonous plants, and the actin promoter is used for rice. However, several studies have shown that foreign gene expression may be enhanced by tissue‐ or organ‐specific promoters. For example, tomatoes transformed via *Agrobacterium* with the cholera toxin‐B subunit (CTB) gene regulated by the CaMV 35S promoter expressed CTB at 0.2%–0.4% of total soluble protein (TSP). By contrast, tomato plants transformed with the same CTB gene but under the control of the E8 tomato fruit‐specific promoter expressed CTB at almost 0.8% of TSP (Guan *et al*., 2013). Low expression level may be due to prokaryotic nature of CTB but expressed via the eukaryotic nuclear genome. So, codon optimization is another approach to maximize the expression of foreign genes. In this approach, codons of the foreign gene are replaced by preferred codons of the host plant. Mason *et al*. (1998) studied the expression of the native and codon‐optimized heat‐labile enterotoxin‐B subunit (LTB) gene from *E. coli*. Expression of the codon‐optimized LTB gene was quite high almost 0.9–12.8 μg/g of tuber and 0.17–1.85 μg/mg of total protein as compared with the native bacterial gene (Mason *et al*., 1998). The use of signal peptides also enhances expression of foreign genes. In plant cells, the endoplasmic reticulum stabilizes foreign proteins and promotes their maturation/processing. It has been suggested that an endoplasmic signal peptide at the C‐terminus of recombinant proteins could enhance expression (Guan *et al*., 2013).

Chloroplast expression system has helped address challenges in low expression level and a number of vaccines antigens against cholera, tetanus, anthrax, plague, polio or canine parvovirus achieving up to 13.17% and 10.11% of TSP in dual cholera and malaria vaccine expressing CTB‐fused apical membrane antigen 1 (AMA1) and merozoite surface protein 1 (MSP1) (Davoodi‐Semiromi *et al*., 2010) and >70% of TSP for CTB–proinsulin (Ruhlman *et al*., 2010). These are a few illustrative examples, but readers referred recent reviews where a more comprehensive list of vaccine antigens or biopharmaceuticals expressed in chloroplasts are provided (Daniell *et al*., 2016a,b; Davoodi‐Semiromi *et al*., 2010; Jin and Daniell, 2015). When eukaryotic human or viral genes are expressed in prokaryotic chloroplasts, expression levels could be very low. However, codon optimization by elimination of rare codons and use of codon usage hierarchy from 130 sequenced chloroplast genomes has resulted in >50‐fold high level expression (Chan *et al*., 2016; Daniell *et al*., 2016a).

### Vaccine delivery systems

#### Parenteral delivery

Syringe and needle‐based injections is the most common method for the administration of vaccines in which vaccines can be delivered through intradermal, intramuscular and subcutaneous route. Vaccines are delivered in the dermis layer in intradermal route, muscular layer below dermis in intramuscular route and fatty tissue between dermis and muscular layer in case of subcutaneous mode of delivery. Immune response in parenteral delivery depends on adopted method for delivery of vaccine antigen. The administration of inactive vaccines in most of animals is very laborious because of intramuscular or subcutaneous injections. Inactive vaccines can produce very low systematic immunity. In humans, needle‐based delivery is associated with infections and inadequate maintenance of cold chain during transportation (Kwon *et al*., 2013).

#### Mucosal delivery

Our digestive tract is covered by mucosal layer, richly supplied with blood vessels that directly enter into jugular vein. Mucosal delivery system directly transports vaccine antigens to immune cells in the mucosal layer and to the blood circulation system. Oral and nasal routes are most common routes for delivery and much more convenient for both humans and animals as compared to injectable mode of delivery (Kwon *et al*., 2013). Amani *et al*. (2011) reported that subcutaneous and orally immunized mice with plant‐derived EspA, Intimin and Tir proteins (EITs) produced significant anti‐EIT IgG and faecal IgA, but no IgA was observed in case of parenteral delivery (Amani *et al*., 2011). In another study, orally immunized mice with CTB (cholera toxin‐B subunit)‐fused malarial antigens (AMA1) and (MSP1) produced antigen‐specific antibodies and showed protection against malarial parasite and cholera toxin challenge (Davoodi‐Semiromi *et al*., 2010), conferring both mucosal immunity and systemic immunity. In a recent study, injected polio vaccine did not generate any IgA, but oral polio vaccine made in plant cells generated both IgA and IgG1 and neutralized all three polio serotypes (Chan *et al*., 2016).

Intranasal delivery is also effective as it is richly supplied by dendritic cells (DCs). Most of live vaccines in animals are delivered as sprays or aerosols. The major drawback of live virus vaccines is that although they are given to animals of a specific age, the aerosol mode of administration can unintentionally inoculate younger or more susceptible animals, which can ultimately cause death (Alexander, 2012; Kwon *et al*., 2013).

#### Oral delivery

Promising results of orally delivered plant‐based vaccines offer a new opportunity to address current challenges in their delivery because gut is the largest surface area for absorption in the body. Moreover, gut immune system is very important to control infections because it acts as the first line of defence against infecting pathogens (Azizi *et al*., 2010). Plant cell wall protects vaccine antigens from degradation by acidic environment in digestive system before it reaches the gut where commensal microbes digest cell wall and release vaccine antigens in the gut lumen (Kwon *et al*., 2013). Antigen uptake across the gut epithelium is facilitated by tags fused to antigens for specific delivery to immune cells (Xiao *et al*., 2016) CTB, LTB or DCs peptides act as carriers for antigen delivery to immune cells. CTB assembles as pentameric structures and binds to GM1 receptors in intestinal epithelial cells. Oral delivery of plant‐based vaccine against different animal diseases has showed promising results. Orally immunized mice with F1V antigen against plague produced high titres of IgG1, IgA and 88% mice were protected after lethal aerosol challenge of *Yersinia pestis* (Arlen *et al*., 2008). In another study, orally immunized mice with plant cells produced CTB‐specific intestinal IgA and serum IgG and showed 100% protection against cholera toxin challenge (Davoodi‐Semiromi *et al*., 2010). Similarly when mice and pigs were orally immunized with E2 glycoprotein against swine flu virus, they produced the E2‐specific systematic, mucosal and cellular immune responses (Jung *et al*., 2014). Similarly, orally immunized mice with H5 of (HPAI) A against bird flu elicited high level of HA‐specific systematic IgG and mucosal IgA, strong Th1 responses together with IgG2b production and 72% mice were protected after viral challenge (Lee *et al*., 2015). Another proof for oral delivery of plant‐based vaccines was observed in orally immunized pigs against porcine reproductive and respiratory syndrome, pigs fed with transgenic plant cells produced antigen‐specific IgA and IgG and neutralizing antibodies (Chia *et al*., 2010). Chan *et al*. (2016) produced oral booster vaccines against poliovirus as WHO approves complete replacement of oral polio vaccine (OPV) with one dose of inactivated poliovirus vaccines. Oral boosting of highly expressed VP1 gene in plant chloroplasts using plant‐based adjuvants, after single priming with IPV, produced high‐titre IgG1 and IgA against VP1 protein, but IPV alone did not produce any IgA. Two doses of IPV or single IPV priming followed by oral boosters resulted in the production of high levels of neutralizing antibodies against all three poliovirus Sabin serotypes. However, single dose of IPV produced low levels of IgG1, neutralizing antibodies but no IgA (Chan *et al*., 2016).

### Oral vaccines against major zoonotic diseases

#### Viral diseases

Rabies is the most common zoonotic infection that circulates among dogs and wild bats. It is a major cause of economic loss in livestock industry and animals get infected by bitting of vampire bats. A number of rabies cases in humans in Latin America have been reported due to bat bitting. Rabies is a major public health concern in developing countries as it caused 55 000 deaths annually according to WHO report and millions of death in animals (Loza‐Rubio *et al*., 2012). Rabies infection spreads through a virus that belongs to the family Rhabdoviridae. Currently available vaccines are satisfactory but requirement for refrigeration at 4 °C and high cost are serious limitations in developing countries. Plant‐based vaccines offer potential solutions to these problems (Loza‐Rubio *et al*., 2012). Transiently expressed nucleoprotein of rabies virus produced high level expression and was immunogenic in mice and conferred protection against rabies viral challenge (Arango *et al*., 2008). Singh *et al*. (2015) fused ricin toxin‐B chain (rgp–rtxB) with this glycoprotein in tomato hairy roots, which produced immune response after intramucosal immunization. The high affinity of CTB to GM1 receptors confirmed its anticholera toxin and antirabies antibodies (Roy *et al*., 2010). Plant‐based rabies vaccines, expressed transiently in spinach, is in phase I clinical trials; five of nine volunteers produced neutralizing antibodies against rabies virus (Takeyama *et al*., 2015).

The swine flu virus belongs to the family Flaviviridae, which causes contagious swine flu disease in pigs and is a major burden in the livestock industry. Influenza is also a major challenge in humans as it has been estimated that millions of people each year are vaccinated against flu. Influenza virus infects farm animals and transmits to humans either by direct contact or through contaminated food. Many cases were reported where pig farmers showed symptoms similar to swine influenza after interaction with infected pigs. In 2009, outbreak of swine influenza caused 17 000 deaths around the world (WHO Situation updates—Pandemic (H1N1) 2009 http://www.who.int/csr/disease/swineflu/updates/en). Many vaccination strategies are in practice to control swine flu fever including live attenuated vaccines but there are some drawbacks of live vaccine including high cost of production, virus inversion and low‐temperature storage requirement. Oral and plant‐based vaccines offer alternative solutions (Jung *et al*., 2014). E2 structural protein expressed in tobacco chloroplasts conferred protective immune response in mice upon oral delivery (Shao *et al*., 2008). Jung *et al*. (2014) also produced transgenic rice calli expressing E2 structural protein and observed protective immune response in orally immunized mice; pigs generated E2‐specific systemic, mucosal and cellular immune responses.

Avian influenza is highly infectious and contagious disease that can cause 100% mortality in livestock. As this disease is usually spread in large farms, massive vaccinations are needed to control disease in birds and animals. Plant‐based vaccines offer the best solution to control this disease in large animal farms (Firsov *et al*., 2015). Expressed HA in endoplasmic reticulum from avian influenza HPA1 resulted in high level expression; immunogenicity of transgenic *Arabidopsis* was confirmed from orally immunized mice with high‐level HA‐specific systematic IgG and mucosal IgA, strong Th1 responses together with IgG2b production and 72% of immunized mice were protected after viral challenge (Lee *et al*., 2015). In 2009, in response to pandemic swine flu, USDA sponsored US$100 million by funding four companies: Fraunhofer USA Center in Delaware, Kentucky Bioprocessing in Owensboro, Medicago USA in North Carolina, Texas A&M University system and G‐Con from Texas, to produce 100 million doses of influenza vaccines. Medicago Inc. developed more than 100 million doses of virus‐like particles (VLP)‐based influenza vaccines against H5 strain that is now undergoing phase II clinical trials and H5N1 transient expression‐based vaccine has completed its phase I and II clinical trials (Rybicki, 2014).

#### Bacterial diseases

Anthrax is the most common emerging zoonotic infection that is spread by the bacterium *Bacillus anthracis*. Anthrax was used as a biological weapon in 2001 and killed five people in the United States. Anthrax outbreaks have been continuously recorded in Asia, Africa and South America. Anthrax is an animal disease, but humans get infected during hunting, through contaminated food or direct contact with animals. Current vaccines against human and animal anthrax are injectable protective antigen obtained from culture filtrate on *Bacillus anthracis*. There are several limitations to this vaccine, including requirement of several boosters (up to eight) and withdrawal of certain batches due to toxin contamination in the culture filtrate. Koya *et al*. (2005) produced transplastomic tobacco by expressing the anthrax protective antigen (PA) and observed protective immune response in immunized mice producing high‐titre IgG antibodies against anthrax (1 : 320 000) and conferred 100% protection after challenge with lethal dose of *Bacillus anthracis*. Protective immune response was confirmed by oral feeding with transgenic plants and challenge with lethal dose of *Bacillus anthracis*. Orally immunized mice produced IgA, IgG1, IgG2a titres and showed 60%–80% protection after challenge (Gorantala *et al*., 2011).

*Yersinia pestis*, a bacterial and zoonotic pathogen, is the causative agent of infectious plague in humans. Plague infection can be bubonic (infection in lymph nodes), septicaemic (infection in blood vessels) or pneumonic (infection in lungs). Plague causes severe infection in humans with 90% mortality if remained untreated. Animals particularly rodents are the main reservoir of *Yersinia pestis*. Humans get infected by rats through fleas. Few recent plague outbreaks have been reported, including those in Asia. Currently available vaccines use live attenuated or killed *Y. pestis* with certain risks; so there is no approved plague vaccines in the United States, even though CDC lists this among biological weapons (category A) (Sinclair *et al*., 2008). Arlen *et al*. (2008) expressed high levels of F1‐V in tobacco chloroplasts and orally fed mice were highly immunogenic and showed 88% protection after *Y‐pestis* lethal challenge. F1‐V expressed in lettuce chloroplasts produced much lower level of antigens but showed immunogenicity (Arlen *et al*., 2008; Rosales‐Mendoza *et al*., 2010).

Tuberculosis (TB) is zoonotic and infectious disease of bacterial origin in both animals and humans. In 2010, TB affected 8.8 million people and resulted in 1.5 million deaths. TB has a high mortality rate as every minute it causes the death of four of twenty infected people. In developed countries, TB has been mostly eliminated but it is still a problem in most of the developing countries. *M. tuberculosis* is causative agent of TB in humans, while bovine acquires TB infection with *M. bovis*. Both species are closely related. *M. bovis* is risky in humans as 10% TB infection in humans is zoonotic due to *M. bovis* (Müller *et al*., 2013). BCG is only available vaccine for tuberculosis but this has several limitations; drug‐resistant TB is yet another emerging challenge. Plant‐based vaccine could offer potential solutions. Transgenic modified carrot with *Mycobacterium tuberculosis* genes *cfp10, esat6* and *dIFN* produced very low levels of antigen (0.035% TSP), and orally immunized mice with transgenic carrot produced both cell‐mediated and humoral immune responses (Permyakova *et al*., 2015). Chloroplast transformation of CTB‐fused ESAT6 and Mtb72F in tobacco and lettuce leaves produced much higher level of expression (up to 7.5% TSP) and lyophilized plant cells could be stored at ambient temperature for several months, thereby eliminating the cold chain and this could facilitate development of an affordable vaccine (Lakshmi *et al*., 2013).

Listeriosis is an infectious zoonotic disease in humans and animals that is caused by the bacterium *Listeria monocytogenes*. This disease is transmitted to humans through contaminated or uncooked food. Infection can be severe in newborn, pregnant women and individuals with weak immune system. Severe complications lead to encephalitis. Listeriosis is a neglected zoonotic pathogen and plant‐based vaccines could offer the best solution for Listeriosis. An attempt was made to produce plant‐based vaccine against Listeriosis in which orally immunized mice with transgenic potato showed very promising results by significantly reducing the bacterial burden in spleen and liver after challenge with *Listeria monocytogenes* (Ohya *et al*., 2005).

Pasteurellosis is also a common infection found in humans and animals that is caused by the bacterium *Pasteurella*. It is responsible for a huge loss in cattle and pig industry, and infection is transmitted to humans by animals bite and contaminated food. An attempt to produce low‐cost edible vaccines against Pasteurellosis resulted in a significant immune response in rabbits fed with plant‐based GS60 (Lee *et al*., 2008).

#### Parasitic diseases

*Toxoplasma gonidii* causes congenital, neurological and ocular Toxoplasmosis in birds, humans and mammals. It is also a zoonotic parasitic pathogen that is transmitted to humans through contaminated food or direct exposure to contaminated soil; infection can be severe among pregnant women and immunocompromised individuals (Chan and Daniell, 2015; Jones *et al*., 2007). GRA4 antigen from *T. gonidii* was expressed in chloroplasts; orally immunized mice with transgenic leaves produced cellular and mucosal immune responses and reduced cyst burden by 60% in mice brain after challenge with *T. gondii* (Yácono *et al*., 2012). In another study, fusion of heat‐shock protein of LiHsp83 to SAG1 enhanced expression of SAG1 in tobacco chloroplasts and reduced cyst load in mice upon oral delivery (Albarracín *et al*., 2015).

Malaria is another devastating diseases spread by Plasmodium falciparum, causing 500 million illness cases, 1 million deaths annually. Currently, there is no licensed vaccine and promising results of plant‐based vaccines against malaria are under development. Davoodi‐Semiromi *et al*. (2010) fused (CTB) *Vibrio cholerae* with malarial vaccine antigens apical membrane antigen‐1 (AMA1) and merozoite surface protein‐1 (MSP1). They observed high level expression (up to 14% TSP) of CTB‐AMA1 and CTB‐MSP1 in lettuce and tobacco chloroplasts. Orally immunized mice with CTB (cholera toxin‐B subunit)‐fused malarial antigens (AMA1) and (MSP1) produced antigen‐specific antibodies and showed protection against malarial parasite and cholera toxin challenge (Davoodi‐Semiromi *et al*., 2010). Jones *et al*. (2013) expressed engineered VLPs fused with Pfs25 and alfalfa mosaic virus coat protein (Pfs25‐CP VLP) in tobacco plants using a tobacco mosaic virus‐based ‘launch’ vector system. Immunization of mice with one or two doses of purified Pfs25‐CP VLPs induced antibodies with transmission blocking activity that was persistent for 6 months postimmunization. Similarly, in another study, Pfs25 gene fused with lichenase (LicKM) carrier was transiently expressed in tobacco plants; immunized mice and rabbits with transgenic tobacco produced transmission blocking antibodies that have been persisted up to 6 months (Jones *et al*., 2015). Table 1 summarizes the plant‐based oral vaccines against zoonotic diseases.

**Table 1:** Vaccines antigens against Zoonotic Diseases expressed in edible plants or tobacco

Diseases	Expressed antigen	Expression system	Expression host	Expression level	Immune response	References
Anthrax (Bacillus anthracis)	PA (protective antigen)	Transplastomic	Tobacco	4.5–18.5 of TSP	Immunized mice produced high‐titre IgG antibodies against anthrax almost 1 : 320 000; 100% protection was observed in immunized mice after challenge with lethal dose of *Bacillus anthracis*.	Koya *et al*. (2005)
	[PA(DIV)]	Transplastomic	Tobacco	5.3% of TSP	Immunized mice produced PA‐specific IgA and IgG. Higher titre of IgG antibodies was observed at 5th bleed up to 2.4 × 105 100% protection was observed in immunized mice after challenge with lethal dose of *Bacillus anthracis*	Gorantala *et al*. (2011)
	PA (protective antigen)	Transplastomic	Tobacco	2.5%–4% of TSP	Immunized mice produced PA‐specific IgA and IgG. Higher titre of IgG antibodies was observed at 5th bleed up to 3.5 × 104 and 7.7 × 104. 100% protection was observed in immunized mice after challenge with lethal dose of *Bacillus anthracis*	Gorantala *et al*. (2014)
	PA (protective antigen)	Transplastomic	Lettuce	7% of TSP	Not done	Rasouli *et al*. (2014)
Rabies Virus	G protein of rabies virus	Transgenic	Carrot	0.4%–1.2% of TSP	Immunized mice produce antibodies against rabies and 66% immunized mice showed protection against virus challenge	Rojas‐Anaya *et al*. (2009)
	G protein fused with CTB	Transient	Tobacco	0.4% of TSP	Not done	Roy *et al*. (2010)
	G protein of rabies virus	Transgenic	Maize	25 μg/g of fresh seed tissue	Immunized sheep with transgenic maize produced antibodies against rabies virus and 50%–83% protection was observed in immunized sheep after virus challenge	Loza‐Rubio *et al*. (2012)
	Rabies virus G protein	Transgenic	Tomato hairy roots	0.9%–1.1% of TSP	Immunized mice with RGP‐RTP produced specific immune response against RGP‐RTP in the form of IgG1, IgG2, TH2 lymphocyte	Singh *et al*. (2015)
Plague	F1‐V	Transplastomic	Tobacco	14.8% of TSP	Orally immunized mice produced high‐titre IgG1, IgG2a, IgA and 88% mice were protected after lethal dose of Y. pestis challenge	Arlen *et al*. (2008)
	F1‐V	Transgenic	Lettuce	0.08% of TSP	Immunized mice produced higher IgG1 and IgG2; no virus challenge assay was performed	Rosales‐Mendoza *et al*. (2010)
	F1‐V	Transgenic	Carrot	0.3% of TSP	Immunized mice produced higher IgG1 and IgG2; no virus challenge assay was performed	Rosales‐Mendoza *et al*. (2011)
Swine Flu Classic Swine Flu Virus (CSFV)	E2 glycoprotein	Transgenic	Lettuce/alfalfa	10 μg/1 g of lyophilized leaves for alfalfa and 160 μg/g of dry leave for lettuce	Serum and faecal pellet from immunized mice confirmed the presence of IgA and IgG	Legocki *et al*. (2005)
	E2 glycoprotein	Transplastomic	Tobacco	1%–2% of TSP	Orally immunized mice did not produce any specific response as compared to subcutaneous immunization which produced CSFV‐specific serum IgG	Shao *et al*. (2008)
	E2 glycoprotein	Cell suspension culture	Rice	5.1 μg/mg of transgenic callus	Orally immunized mice and pig had the E2‐specific systematic, mucosal immune responses and cellular immune response was also observed in the form of different cytokines	Jung *et al*. (2014)
Bird flu Avian Influenza Virus (AIV)	NA gene of H1N1	Transgenic	Lettuce	0.018%–0.045% of TSP	Orally immunized mice produced significant anti‐NA antibodies at third booster; no virus challenge assay was performed	Liu *et al*. (2012)
	M2e Peptide of H5N1	Transgenic	Duckweed plant	0.12%–1.96% of TSP	Not done	Firsov *et al*. (2015)
	H5 of (HPAI) A	Transgenic	Arabidopsis	700 μg/g (dry weight) or 140 μg/g (fresh leaf) by ER targeting	Orally immunized mice elicited high level of HA‐specific systematic IgG and mucosal IgA, strong Th1 responses together with IgG2b production was observed, and 72% protection was observed to immunized mice after virus challenge	Lee *et al*. (2015)
	NP of H3N2	Transgenic	Maize	8.0–35 μg/g of corn seed	Production of IgA, IgG, IgG2, TH1, TH2 from immunized mice confirmed systematic, mucosal and cell‐mediated immune responses	Nahampun *et al*. (2015)
Rotavirus	VP6 gene	Transient	Chenopodium leaves	0.25% of TSP or 1.54 μg/g of fresh leaves	Orally immunized mice produced anti‐VP6‐specific serum IgG and significant increase in titre of saliva IgA was observed. 60% mice protection was observed after virus challenge	Zhou *et al*. (2010)
	C486 BRV VP8 protein	Transplastomic	Tobacco	600 μg/g of fresh tissue	High titre of IgG antibodies specific to Vp8 was observed from sera of immunized mice, and 80%–100% protection was observed in newly born mice from immunized female after rotavirus challenge.	Lentz *et al*. (2011)
	MucoRice‐ARP1(heavy chain antibody fragment)	Transgenic	Rice	11.9% of TSP	Administration of MucoRice‐ARP reduced the symptoms of disease, and neutralization assay performed in MA104 cells showed complete protection against rotavirus infection.	Tokuhara (2013)
Tuberculosis (*Mycobacterium tuberculosis*)	Ag85B, MPT83, MPT64, ESAT6	Transgenic	Potato	Not reported	Immunized mice produced high‐titre IgG and IgA antibodies against antigens, and stimulated CD4+ and CD8+ led to increased production of Th cells and cytokines	Zhang *et al*. (2012)
	ESAT6 and CFP10	Transgenic	Carrot	0.002%–0.056% of TSP	Immunized mice elicited both cell‐mediated and humoral immune responses	Uvarova *et al*. (2013)
	CFP10, ESAT6, dIFN	Transgenic	Carrot	0.035% of TSP	Immunized mice confirmed both cellular and humoral immune responses	Permyakova *et al*. (2015)
Brucellosis	U‐Omp19	Transient	Tobacco	Not reported	Immunized mice produced specific response in the form of CD4+ T cells, IL17 and protected against a mucosal challenge with Brucella abortus	Pasquevich *et al*. (2011)
Toxoplasmosis	Gra4	Transplastomic	Tobacco	0.2% of TSP	Orally immunized mice produced cellular and mucosal immune response	Yácono *et al*. (2012)
	SAG1	Transient	Tobacco	0.1–1.3 μg/g of Fresh weight	Immunized mice with transgenic SAG1 leaf extracts were protected against cyst challenge, and production of Th1 and IFNγ from immunized mice confirmed humoral and cellular immune response	Laguía‐Becher *et al*. (2010)
	SAG1	Transplastomic	Tobacco	0.1–0.2 μg/g of Fresh weight	Orally immunized mice with transgenic LiHsp83‐SAGI reduced 57% cyst burden after T.gondii challenge	Albarracín *et al*. (2015)
Psiattacosis	LTB‐fused MOMP gene	Transgenic	Rice	0.0033%–0.0054% of TSP	Not performed	Zhang *et al*. (2008)
	MOMP gene	Transgenic	Rice	Not reported	Orally immunized mice produced serum IgG and faecal IgA against MOMP, Production of cytokines IL‐2, IL‐4, IL‐5, TGFβ, IFNγ confirmed cellular immune esponse, and 50% were protected against lethal dose of *Cp.psittaci*	Zhang *et al*. (2013)
Gastroenteritis Transmissible Gastroenteritis Virus (TGEV)	S protein of TGEV	Transgenic	Corn	0.1%–0.8% of TSP	Immunized pig produced IgA, IgG and neutralizing antibodies	Lamphear *et al*. (2004)
	SIP	Transient	Tobacco	2% of TSP	Neutralizing antibodies were confirmed *in vitro* on mammalian cell and in *in vivo*, immunized pigs produced 60%–70% neutralizing antibodies	Monger *et al*. (2006)
Crimean‐Congo Haemorrhagic Fever Virus (CCHFV)	G1&G2	Transgenic	Tobacco	0.45% of TSP in hairy roots	Orally immunized mice produce G1/G2 specific IgG with titre range (1 : 65 536) and faecal IgA with titre range (1 : 512)	Ghiasi *et al*. (2011)
Japanese Encephalitis Virus Tremblay *et al*. (2010)	E protein	Transgenic	Rice	1.1–1.9 μg/mg of TSP	Production of JEV‐specific IgA and IgG confirmed mucosal and humoral immune responses	Wang *et al*. (2009)
West Nile Virus	VLPs from Norwalk virus and (mAbs) form West Nile and Ebola viruses	Transient	Lettuce	0.23–0.27 mg/g of fresh leave weight	Immune studies not performed but focussed on reduction in neutralizing assay confirmed neutralizing antibodies against WNV	Lai *et al*. (2012)
Pasteurellosis	GS60	Transgenic/Transient	Alfalfa/Tobacco	0.02% of TSP for transgenic plants	Immunized rabbit with transgenic alfalfa produced antibodies against GS60	Lee *et al*. (2008)
(Listeriosis) Listeria monocytogenes	IFN‐α	Transgenic	Potato	Not reported	Orally immunized mice with transgenic IFN‐α showed its ability to reduce bacterial burden as compared to nontransgenic plants	Ohya *et al*. (2005)
Newcastle Disease Virus (NDV)	HN gene	Transgenic	Tobacco	0.069% of TSP	anti‐HN serum IgG, no virus challenge study was performed	Hahn *et al*. (2007)
	F and HN	Transgenic	Potato	0.3–0.6 μg/mg of TSP	NDV‐specific IgA and IgG	Gómez *et al*. (2008)
	HN gene	Transgenic	*Centella asiatica*	Not reported	Not done	Song Lai *et al*. (2012)
	HN ectodomain	Transgenic	Tobacco	0.2%–0.4% of TSP	Immunized mice produced NDV‐specific antibodies	Lai *et al*. (2013)
	F and HN epitope	Transient	Tobacco	Not reported	Not reported	Shahriari *et al*. (2015)

#### Oral vaccines against nonzoonotic animal diseases

Health of animals is very important for security of humans as 71% of emerging diseases in humans are due to zoonotic pathogens. A number of successful efforts have been made to produce plant‐made veterinary vaccines against nonzoonotic pathogens that are quite severe in animals, leading to major losses in livestock industry. Foot‐and‐mouth disease is common in bovine, horse, sheep, pig and goat that cause high fever, weight loss and erosions in feet and mouth. This is a major challenge in livestock economy by reducing milk and meat production (Ruiz *et al*., 2015). Rao *et al*. (2012) produced a bivalent vaccine against foot‐and‐mouth diseases expressing VP1 structural protein from two serotypes A and O. Oral feeding of pig with transgenic plants produced specific humoral immune response. Sera from immunized pigs when challenged with FMDV neutralized the virus with no cytopathic effect on BHK‐21 cells (Rao *et al*., 2012). In another approach, VP1 protein was expressed in tobacco chloroplasts (Lentz *et al*., 2010). Several attempts have been made to express BVDV in tobacco (Nelson *et al*., 2012), alfalfa (Aguirreburualde *et al*., 2013) and Panax ginseng (Gao *et al*., 2015) and their immune studies in different animal models showed promising results.

A number of plant‐based vaccines have also been made against different animal diseases. Pig oedema causes heavy economic loss and mortality. It mostly happens in piglets after weaning and its neurological disorder cause sudden death of piglets. Currently, no vaccine is available to protect pigs from oedema diseases. Expressed antigens in plants against pig oedema disease offer potential solutions. Orally Immunized mice and pigs with tobacco seeds expressing Vt2e‐B and FedA conferred protective immune response against O138 *E. coli*, and immunized pigs showed protection against O138 *E. coli* infection (Rossi *et al*., 2014). Antigen expressed in tobacco (Chia *et al*., 2010) and banana (Chan *et al*., 2013) against porcine reproductive and respiratory syndrome (PRRSV) showed promising results.

Poultry is a major industry but unfortunately its growth is severely threatened by a number of pathogens of both zoonotic and nonzoonotic origin. Currently worldwide poultry production is almost 86 million tons and it is likely to increase to 94 million tons in the near future. Currently emerging diseases in field of poultry also lead to development of plant‐based vaccines against different poultry diseases. NDV, infectious bursal diseases, avian influenza, coccidiosis are most severe diseases in poultry. Infectious bursal disease is the most important disease in young chickens that causes high mortality. Currently available vaccines are live and killed virus, which are protective but associated with some disadvantages. VP2 is most selected antigen in plant‐based vaccine against infectious bursal disease virus (IBDV) due to its immunogenic and virus neutralizing ability. Edible vaccines against IBDV have been developed in tobacco (Chen *et al*., 2012; Gómez *et al*., 2013) and rice (Wu *et al*., 2007). Immunized chickens not only produced antibodies against IBDV but also showed protection against IBDV challenge.

Canine parvovirus causes infection in dogs and particularly in puppies. The importance of dog cannot be denied among domesticated animals. Currently live and killed virus‐based vaccines offer the only solution to treat dogs with CPV infection. Live vaccines are always associated with pathogen of low virulence that can be hazardous for dogs with weak immune systems. Many efforts in the field of plant‐based vaccines have been made to find alternate solution to treat or prevent CPV in dogs. Immunization of mice with CTB‐2L21 and GFP‐2L21 peptide from (CPV) VP2 protein (Molina *et al*., 2004) and 2L21‐TD (Ortigosa *et al*., 2010) expressed in chloroplasts generated high titres of IgA, IgG1 antibodies against viral VP2 protein. In another approach, antigens expressed in *Arabidopsis* against rabbit haemorrhagic disease virus (RHDV) (Gil *et al*., 2006) and in tobacco against cottontail rabbit papillomavirus (CRPV) (Kohl *et al*., 2006) showed immunogenicity and promising results. Table 2 summarizes the plant‐based vaccines against nonzoonotic infections.

**Table 2:** Vaccine antigens against Animals Diseases expressed in edible plants or tobacco

Diseases	Expressed antigen	Expression system	Expression host	Expression level	Immune response	References
Expressed Antigens in plants against Bovine and Horse Diseases						
Foot‐and‐Mouth Disease virus (FMDV)	VP1 protein	Transplastomic	Tobacco	51% of TSP	Orally immunized Balb/c mice produced anti‐VP1 FMDV‐specific antibodies	Lentz *et al*. (2010)
	Two serotypes of VP1, O‐ and Asia 1‐type	Transgenic	Maize	Not done	Not reported	Zhang *et al*. (2011)
	Polypeptide P1 gene	Transgenic	Rice	0.6–1.3 μg/mg of TSP	FMDV‐specific IgG and IGA were detected, and 20%–40% mice showed clearance of virus after virus challenge	Wang *et al*. (2012)
	Two serotypes of VP1, O‐A	Transgenic	Sunn hemp plants	1–12 μg/g of TSP	Immunized guinea pigs confirmed FMDV specific humoral immune response, and specific virus challenge study in BHK‐21 cells produced neutralizing antibody against FMDV challenge	Rao *et al*. (2012)
	VP1 capsid protein	Transient	Tobacco	Not reported	Not reported	Habibi‐Pirkoohi *et al*. (2014)
Bovine Viral Diarrhoea Virus (BVDV)	E2 glycoprotein	Transient	Tobacco	600 μg/g of fresh leave	Sera of Immunized guinea pig were tested by indirect ELISA, which produced high titres of anti‐BVDV‐specific antibodies. Neutralization assay from guinea pig sera in MDBK cells showed the production of neutralizing antibodies after BVDV challenge	Nelson *et al*. (2012)
	E2 glycoprotein	Transgenic	Alfalfa	1 μg/g of fresh leave weight	Immunized guinea pig produced high titre of neutralizing antibodies almost more than 2.4 against BVDV; incubated MDBK cells with sera from immunized guinea also confirmed viral antigen after virus neutralization assay	Aguirreburualde *et al*. (2013)
	E0 glycoprotein	Transgenic	Astragalus	Not reported	Immunized deer produced humoral and cell‐mediated immune responses against BVDV.	Gao *et al*. (2014)
	Erns glycoprotein	Transgenic	Panax ginseng	Not reported	Immunized deer produced humoral and cell‐mediated immune responses against BVDV	Gao *et al*. (2015)
Peste des Petits Ruminant Virus (PPRV)	HN protein	Transgenic	Peanut plant	Not reported	Immunized sheep with transgenic leaves produced HN‐specific antibodies against PPRV and serum from immunized sheep reacted and showed proliferation in PPRV‐infected VERO cells as compared to control	Khandelwal *et al*. (2011)
Expressed Antigens in Plants against Pig Diseases						
Pig oedema Diseases	Stx2EB	Transgenic	Lettuce	80 mg per 100 g fresh weight	Not done	Matsui *et al*. (2011)
	Vt2e‐B and F18	Transgenic	Tobacco	0.3% of TSP of 0.6 mg seeds	Immunized mice with tobacco seeds confirmed the increased numbers of mucosal IgA‐producing cells by immunohistochemistry of small and large intestines	Rossi *et al*. (2013)
	Vt2e‐B and FedA subunit of F18	Transgenic	Tobacco	Not done	Orally immunized mice with FedA and VT2eB produced protective immune response against *E. coli* strain. The immunized pigs appeared preventing the symptoms of disease after challenge with O138 *E. coli* strain	Rossi *et al*. (2014)
*E. coli*‐mediated Diarrhoea in Pigs (ETEC)	faeG gene	Transplastomic	Tobacco	0.15% of TSP	Orally immunized mice produced FaeG‐specific antibodies. Moreover, sera from immunized mice confirmed neutralizing effect from ETEC in rabbits by ideal loop assay	Shen *et al*. (2010)
Porcine Epidemic Diarrhoea Virus (PEDV)	CTB‐fused COE	Transgenic	Lettuce	0.0065% of TSP	Not done	Huy *et al*. (2011)
	M cell‐fused COE	Transgenic	Rice	0.083% of TSP	Isolated lymphocyte from spleen and Peyer's patches of immunized mice confirmed three‐ to four‐fold higher level of COE‐specific IgA and IgG as compared to wild type	Huy *et al*. (2012)
Porcine Reproductive and Respiratory Syndrome Virus (PRRSV)	ORF5	Transgenic	Tobacco	0.011% of TSP	Orally immunized pigs confirmed significant amount of anti‐PRRSV IgG from sera and IgA from saliva. Serum from immunized pigs also confirmed neutralizing antibodies at titre of 1 : 8	Chia *et al*. (2010)
	ORF5	Transgenic	Banana	0.021%–0.037% of TSP	Immunized pigs by oral feeding of transgenic banana leaves produced strong serum and saliva anti‐PRRSV response. Virus challenge to immunized pigs decreased viral load in tissues by producing neutralizing antibodies	Chan *et al*. (2013)
Expressed Antigens in Plants against Poultry Diseases						
Coccidiosis	EtMIC2 from Eimeria tenella	Transient	Tobacco	Not reported	Immunized birds produced high‐titre serum IgG with an average 940 on 21st day and 1340 on 28th day. Partial protection in birds was observed after challenge with virulent *E.tenella* oocytes	Sathish *et al*. (2011)
	EtMIC1 and EtMIC2 from Eimeria tenella	Transient	Tobacco	25 mg/kg of fresh biomass	Immunize birds with bivalent formulation produced better systematic immune response and better protection against challenge as compared to monovalent formulation	Sathish *et al*. (2012)
Infectious Bursal Disease Virus (IBDV)	VP2 antigen from IBDV	Transient	Tobacco	Not reported	Immunized chicken produced high‐titre IBDV‐specific IgG and were protected from IBDV challenge	Chen *et al*. (2012)
	VP2	Transient	Tobacco	1% of TSP	Intramuscularly immunized chicken with transgenic tobacco elicted specific humoral response and neutralizing antibodies	Gómez *et al*. (2013)
Expressed Antigens in Plants against Dog diseases						
Canine Parvovirus (CPV)	CTB‐fused 2L21 and GFP‐fused 2L21	Transplastomic	Tobacco	31% of TSP for CTB‐2L21 and 22.6 of TSP for GFP‐2L21	Immunized Balb/c mice with leaf extract from CTB‐fused 2L21 and GFP‐fused 2L21 produced anti‐2L21 antibodies with titre range from 200 to 25 000, and anti‐VP2 response against viral VP2 protein confirmed its protective immune response against CPV	Molina *et al*. (2004)
	CTB‐fused 2L21	Transplastomic	Tobacco	31% of TSP	Immunized mice with CTB‐2L21 produced high‐titre IgG and IgA with ability to recognize viral protein VP2	Molina *et al*. (2005)
	VP2 gene	Transgenic	Tobacco	Not done	Not done	Xiong *et al*. (2008)
	2L21‐TD (Tetramerization domain p53)	Transplastomic	Tobacco	6% of TSP	Immunized mice with 2L21‐TD produced high‐titre antibodies and its ability to recognize viral protein VP2 confirmed the antigenicity of 2L21‐TD	Ortigosa *et al*. (2010)
Expressed Antigens in Plants against Rabbit Diseases						
Rabbit Haemorrhagic Disease Virus (RHDV)	VP60	Transient	Arabidopsis	0.3%–0.8% of TSP	Immunized mice with transgenic crude extracts produced specific antibodies against VP60 with titre range between 1 : 10 000 and 1 : 32 000	Gil *et al*. (2006)
	VP60	Transgenic	Multiple plants	0.01%‐0.7% of TSP	Immunized rabbits produced anti‐VP60‐specific antibodies and protected from RHDV challenge.	Mikschofsky *et al*. (2009)
Cottontail Rabbit Papillomavirus (CRPV)	L1 capsid protein	Transgenic/Transient	Tobacco	1.0 mg/kg for transgenic and up to 0.4 mg/kg for transient	Immunized rabbits produced specific antibodies against L1 capsid protein and were protected from virus challenge, but *in vitro* assay did not show any neutralization efficiency	Kohl *et al*. (2006)
Edible Vaccines against Aquaculture						
*E. coli* (Aquaculture)	LTB fused to GFP, VP from Canine parvovirus, HA from Influenza virus	Transgenic	Potato tubers	Not reported	Oral feeding of fish by plant‐based fusion protein produced specific systematic humoral immune response.	Companjen *et al*. (2006)

### Current facilities for the production of plant‐based vaccines

The recent advancement and successful approaches to develop plant‐derived vaccines have attracted attention for creating large commercial facilities to produce large quantities of vaccine antigens under good manufacturing practice (cGMP) standards. The first set‐up for commercial‐ and large‐scale production of plant‐derived vaccines was developed in 1999 and was based on transient expression systems. After that, extensive progress was made in the field of plant vaccines: high‐level expression was achieved and the concepts of plant virus vectors and the vacuum infiltration process were introduced. In 2007, the Defense Advanced Research Project Agency (DARPA) funded a project for the development of cGMP manufacturing units for large‐scale, rapid production of plant‐derived vaccines. After realizing that low‐cost, plant‐derived vaccines are a better tool to control many infectious diseases in humans, DARPA financed projects at Kentucky BioProcessing (Owensboro, KY), Medicago (Durham, NC) and Caliber Bio Therapeutics (Bryan, TX) for the development of cGMP facilities for plant‐made vaccines. Caliber Biotherapeutics is currently the largest manufacturing facility and can produce 3500 kg of plant material at one time under LED illumination system, automatic hydroponic facility and high‐throughput infiltration system. LED illumination system has reduced growth time and energy cost. Plants after 5 weeks of seeding in automated hydroponic system under constant LED light are moved to the infiltration area, which is 18.3‐metre‐long vessel with ability to hold 7000 L infiltrate. After infiltration, plants are placed on postinfiltration growth racks for 6‐10 days, then transported to harvest area and finally driven to downstream area for final purification. As a world's largest plant‐based manufacturing facility, it produced more than 100 million doses of H1N1 influenza subunit vaccines for phase I clinical trials. This investment in cGMP manufacturing units is a major advance in the field of plant‐based vaccines and will permit the production of large quantities of vaccines at the time of an outbreak (Holtz *et al*., 2015).

## Conclusions

Major efforts have been made during last decade to use plants to express foreign antigens against various animal and human diseases. The success of the first commercial plant‐made vaccine against NDV by Dow Agro Sciences and FDA approval of biopharmaceutical to treat Gaucher's disease produced in carrot cells offer hope for rapid commercialization of plant‐made vaccines. Moreover, successful human trials of plant‐based vaccines against norovirus, influenza, rotavirus, rabies, *E. coli* and HBV infections also contribute to this growing industry. Plant‐made vaccines possess all the desirable attributes of vaccines including long‐term storage and stability at room temperature. But the main challenge in plant‐based vaccines is the need for oral priming with adjuvants. So, new techniques/concepts are needed to overcome this challenge.

## Conflict of interest statement

Henry Daniell, as a pioneer in the field of chloroplast genetic engineering, has several patents in this field but has no financial conflict of interest to declare.
